# A randomized study on migration of the Spectron EF and the Charnley flanged 40 cemented femoral stems: secondary analysis of a randomized controlled trial using radiostereometric analysis (RSA) with 10 years’ follow-up

**DOI:** 10.2340/17453674.2026.46836

**Published:** 2026-11-12

**Authors:** Håkon Greve JOHANNESSEN, Geir HALLAN, Ove FURNES, Anne Marie FENSTAD, Stein Håkon LYGRE, Kristin HAUGAN, Thomas KADAR, Mona BADAWY, Kari INDREKVAM, Arild AAMODT, Paul Johan HØL

**Affiliations:** 1 Department of Clinical Medicine, Faculty of Medicine, University of Bergen, Bergen; 2 The Norwegian Arthroplasty Register, Department of Orthopaedic Surgery, Haukeland University Hospital, Bergen; 3 Coastal Hospital in Hagavik, Department of Orthopaedic Surgery, Haukeland University Hospital, Bergen; 4 Department of Occupational Medicine, Haukeland University Hospital, Bergen; 5 Department of Orthopaedic Surgery, St. Olavs Hospital, Trondheim; 6 Department of Physical Medicine and Rehabilitation, Haukeland University Hospital, Bergen; 7 Department of Orthopaedic Surgery, Lovisenberg Diakonal Hospital, Oslo; 8 Department of Orthopaedic Surgery, Biomatlab, Haukeland University Hospital, Bergen, Norway

## Abstract

**Background and purpose:**

Our primary aim was to compare the 10-year migration of the proximally roughened Spectron EF stem with the Charnley flanged 40 stems in cemented THA. Secondary aims were to assess the influence of polyethylene type on Spectron EF migration, and clinical outcomes.

**Methods:**

In this randomized trial (NCT00698672), 150 patients (70% women; mean age 70 years) underwent cemented THA with either a Charnley stem (n = 30) or a Spectron EF stem (n = 120, 4 groups with different articulating surfaces). The articulations utilized conventional polyethylene (CPE) or highly crosslinked polyethylene (XLPE) cup liners combined with either cobalt-chromium (CoCr) or Oxinium (OxZr) femoral heads. RSA was performed postoperatively and up to 10 years. Migration was analyzed using linear mixed-effects models. Clinical outcome was assessed with the Harris Hip Score (HHS).

**Results:**

After 10 years, 48 patients (14 Charnley, 34 Spectron EF) had evaluable RSA data. Both stems showed posterior translation, subsidence, and retroversion. Retroversion was greater for the Charnley stem than the Spectron EF stem at 3.2° (95% confidence interval [CI] 2.7–3.8) and 1.5° (CI 1.2–1.8), respectively, with a mean difference of 1.7° (CI 1.1– 2.4). The Spectron EF stem had more subsidence at 0.72 mm (CI 0.56–0.88) vs 0.32 mm (CI 0.06–0.57) for the Charnley stem, with a difference of 0.41 mm (CI 0.11–0.71). Spectron EF stems articulating with XLPE had 0.44 mm (CI 0.12–0.76) more subsidence than stems articulating with CPE. HHS improved from 41 (SD 15) to 86 (SD 19) for the Charnley stem and 45 (SD 13) to 86 (SD 15) for the Spectron EF stem with no statistically significant difference between the groups.

**Conclusion:**

After 10 years, the Spectron EF stem showed less retroversion, but more subsidence than the Charnley stem, with otherwise similar migration and clinical outcomes. XLPE articulating stems had more subsidence than CPE with the Spectron EF stem.

Loosening of femoral stems in cemented total hip arthroplasty (THA) is a complex process with multiple contributing factors [1,2]. One identified factor is the impact the bonding between the stem and the cement mantle has for the long-term success of the implant but also the articulation may play a role [3,4]. 2 main principles exist for cemented stems: composite beam, where the stem should be fixed to the cement, or taper slip, which allows the stem to subside as a wedge into the cement. Studies have shown different benefits for these 2 principles with less aseptic loosening and more periprosthetic fractures for the taper slip stems compared with the composite beam stems [5,6]. Regarding articulations, a recent study showed no significant advantage of OxZr over CoCr heads in reducing wear or migration. XLPE demonstrated a major reduction in wear as well as a reduction in cup migration compared with CPE [7].

The satin-finished Spectron femoral stem showed promising results compared with the Charnley stem [8,9]. A new model, the Spectron EF, with a roughened proximal surface, was introduced in 1989 to improve stem–cement bonding. In 2007, this stem combined with the Reflection All-Poly cup was the most used cemented THA in Norway [10]. 7-year survival was similar for the satin-finished Spectron and the new Spectron EF in the Swedish Arthroplasty Register [9].

This study was initiated due to the lack of clinical reports for the proximally roughened Spectron EF stem. In addition, there are few RSA studies of THA that highlight the migration patterns of the femoral stems with long-term follow-up. The 2-year migration data for the stems has already been published, and this is a secondary analysis with 10-year follow-up [11]. The primary aim of this study was to compare the degree of migration of the Spectron EF stem and the Charnley stem at 10 years. Secondary aims were to evaluate the influence of type of polyethylene (PE) used (conventional PE [CPE] or highly cross-linked PE [XLPE]) on the migration of the Spectron EF stem, and to compare clinical outcomes between groups.

## Methods

### Study design

This study is a secondary analysis of a randomized controlled trial using radiostereometric analysis (RSA) with 10 years’ follow-up.

Patients were included between November 2004 and June 2007. Written informed consent was obtained from all participants prior to inclusion. In cases with bilateral disease, only 1 hip was included in the study. Exclusion criteria were a body mass index (BMI) > 35, uncompensated cardiopulmonary disease, malignant disease, dementia, rheumatoid arthritis, or other severe systemic conditions.

Inclusion was only done if investigation on preoperative pelvic radiographs with templates deemed that both implant systems could restore hip biomechanics sufficiently.

The study was reported in accordance with the CONSORT guidelines for multi-arm parallel-group randomized trials, and the revised RSA reporting guideline was also consulted [12,13].

### Intervention

This study is part of a larger study investigating wear and migration of 5 different articulations in THA, utilizing the Charnley and Spectron EF femoral stems [11,14].

Patients were randomized to receive 1 of the 5 articulations. Following randomization, 30 patients were allocated to the Charnley group, whereas 120 patients were distributed across the 4 Spectron EF groups. The 4 groups of Reflection/Spectron EF THAs differed in type of polyethylene (conventional UHMWPE or XLPE) and femoral head material (CoCr or Oxinium). For the primary analysis in the present study (stem migration), the 4 Spectron EF groups were merged into a single group. In addition, supplementary analyses were performed in which the Spectron EF group was stratified according to the type of PE used in the acetabular socket. 8 consultants and 1 resident orthopedic surgeon were responsible for conducting or supervising the operations. The surgeons operated on an equal number of patients in each group. The patients were blinded to their group. The randomization process has been described in greater detail previously [11].

The patients received 1 of the following cemented femoral stems in THAs (Supplementary Figure S1):

1. Charnley flanged 40 monoblock, oval, stainless steel, vaquasheen surface (Ra 0.8 µm) femoral stem with a 22.2 mm head. It articulated with a cemented Charnley Ogee UHMWPE acetabular cup, which was γ-sterilized in nitrogen (DePuy International Ltd, Leeds, UK).
2. Spectron EF modular, square, matte distal surface (Ra 0.7 µm), collared cobalt-chromium femoral stem with a grit-blasted roughened surface (Ra 2.8 µm) on the proximal third of the stem used with either a head of CoCr or Oxinium, articulating with a cemented Reflection All-Poly cup with either CPE or XLPE.

Both these stems use the composite-beam principle for fixation, in which solid bonding between the stem and cement is the goal [3].

The direct lateral approach without trochanteric osteotomy was used for all cases. A third-generation cementing technique was employed in all patients, including entry into the femoral canal via the fossa piriformis, high-pressure lavage, use of a femoral cement restrictor, retrograde cement injection with a cement gun, and cement pressurization. Initially, all components were implanted using Palacos R bone cement with gentamicin (Schering-Plough; Labo N.V., Heist-op-den-Berg, Belgium). From August 2005 onward, Palacos R+G cement (Heraeus Kulzer GmbH, Hanau, Germany) was used. The bone cement was stored at 8° C under refrigerated conditions and removed immediately prior to use. Cement mixing was performed using the Optivac system (Biomet, Warsaw, IN, USA). The femoral stem was inserted 5 minutes after cement mixing, following a 1-minute cement pressurization period. Additional details of the surgical technique have been reported previously [14].

Both femoral components were supplied by the manufacturer with tantalum markers. The Charnley flanged 40 stem was equipped with 2 tantalum markers, and the center of the femoral head was used as a 3rd reference point for calculating stem migration. The Spectron EF stem was supplied with 3 tantalum markers; 1 of these was mounted on a cone that was impacted into a predrilled hole in the femoral neck after implantation. In addition, 4 tantalum markers (1.0 mm in diameter) were inserted into the greater trochanter, 1 marker was placed medially in the calcar region, and 1 marker was inserted into the lesser trochanter (Supplementary Figure S2).

### Outcomes

The initial RSA assessment was performed at a median of 11 days following surgery (interquartile range, 9–15 days). Subsequent RSA evaluations were scheduled at 3, 6, 12, 24, 60, and 120 months postoperatively. All examinations were conducted by the same radiographer to ensure consistency. The current guidelines for RSA studies were consulted, and the technique was standardized accordingly [13]. A uniplanar RSA method was applied, using a calibration cage positioned below the examination table (cage 43; RSA Biomedical, Umeå, Sweden). During image acquisition, patients were positioned supine. Paired radiographic images were obtained simultaneously using a gantry-mounted X-ray tube in combination with a portable X-ray unit. Image acquisition was performed with high-resolution digital plates (Agfa CR MD 4.0; Agfa-Geveart, Mortsel, Belgium), and image digitization was carried out using the ADC Compact digitizer (Agfa).

If 3 or more tantalum markers could not be identified within the same bone segment during repeated RSA examinations, migration and rotation could not be reliably analyzed. The condition number (CN) is a measure of the configuration of the markers in the segment. The mean error of rigid body fitting (ME) is a measure of marker stability. The upper limit on the ME was set to 0.35 mm, and the upper limit for the CN was set to 150 [13]. Translational and rotational movements were calculated along and about the horizontal (x), longitudinal (y), and sagittal (z) axes using signed values. All calculations were performed with UmRSA Digital Measure software, version 5.0 (RSA Biomedical). All hips were normalized to the right side, as this was chosen as reference. Our primary outcome was the translation and migration in all axes of the stem markers in relation to the femoral markers at the 10-year follow-up, with emphasis on subsidence, defined as distal migration along the y-axis, posterior translation along the z-axis, and retroversion, defined as posterior rotation around the y-axis (see Supplementary Figure S2). The precision of the RSA measurements has been described previously [11].

The Harris Hip Score (HHS) was included as a secondary outcome measure to evaluate clinical performance by comparing preoperative values with postoperative scores obtained at the same time points as the RSA assessments. This is a clinical score evaluating hip function, with a scale from 0–100, with 100 indicating optimal function [15]. All HHS evaluations were performed by either a surgeon or a physiotherapist. Both RSA-derived data and clinical outcome measures were analyzed according to the intention-to-treat principle.

### Statistics

Based on an a priori power calculation, a sample size of 30 participants per group was selected [11,14]. Demographic characteristics are reported with range or standard deviation (SD) where relevant. For the RSA data we used linear mixed effects models to estimate the mean values of each outcome for each articulation at each time point, including an interaction term between articulation and time. To account for repeated measurements within individuals, we included a random intercept for each participant. The models were estimated using restricted maximum likelihood (REML) and included all available observations under the missing-at-random assumption [16].

Pairwise comparisons of stem translation and rotation were performed at the 2-, 5-, and 10-year follow-up. No adjustments were made for multiple testing.

Clinical outcomes measured with the Harris Hip Score (HHS) were compared between groups using the Mann–Whitney U test, while within-group changes in HHS from the preoperative assessment to 10 years postoperatively were evaluated using the Wilcoxon signed-rank test. All statistical analyses were 2-sided, with the threshold for statistical significance set at 0.05. Statistical analyses were conducted using SPSS version 29 (IBM Corp, Armonk, NY, USA) and STATA version 18 (StataCorp, College Station, TX, USA) for the linear mixed-effects modeling. Figures were generated using R version 4.5.0 (R Foundation for Statistical Computing, Vienna, Austria).

### Ethics, registration, data sharing plan, use of AI, funding, and disclosures

The trial was registered at ClinicalTrials.gov (NCT00698672) and received approval from the Regional Committee for Medical and Health Research Ethics in Western Norway (REK No. 2014-02370). Deidentified data may be made available upon reasonable request. ChatGPT (OpenAI, San Francisco, CA, USA) was used to assist in shortening and reformulating portions of the abstract and methods section. It was not used to interpret data or to formulate discussion points. The study was supported by funding from OrthoMedic AS, Smith & Nephew Norway AS, and the Regional Health Authority of Western Norway. The first author received a grant from the University of Bergen through the medical student research program. None of the funding bodies had any influence on the study design, data collection, analysis, interpretation of results, or manuscript preparation. Regarding potential conflicts of interest, GH has received speaker honoraria from OrthoMedic, Heraeus Medical, and LINK Norway. OF has received honoraria for lectures on cementing techniques in knee arthroplasty from Heraeus Medical and OrthoMedic, and PJH has received lecture fees from Heraeus Medical. Complete disclosure of interest forms according to ICMJE are available on the article page, doi: 10.2340/17453674.2026.46836

## Results

150 patients (70% women) with a mean age of 70 years (range, 59–80 years) were enrolled to undergo total hip arthroplasty (THA) for primary or secondary hip osteoarthritis (Figure 1, Table 1). At the 10-year follow-up, 19 patients had died, and another 34 had withdrawn their participation due to poor health or for other practical reasons (Figure 1). 14 patients were surgically revised before the 10-year follow-up (2 for dislocation, 5 for infection, and 7 for aseptic loosening of either or both components) and were therefore excluded from further RSA examinations after the revision. Furthermore, 4 patients in the Charnley group and 31 patients in the Spectron EF groups were excluded from stem-migration assessment due to technical issues with the RSA measurements such as high CN, high ME, or the loss of tantalum markers. Thus, 48 patients, 14 in the Charnley group and 34 in the Spectron EF groups, had available RSA measurements for analysis at 10 years.

**Table 1:** Cemented total hip arthroplasty with 2 different stems: demographics of patients at baseline. Values are count or mean with (range)

Characteristic	Charnley flanged 40 (n = 30)	Spectron EF (n = 120)
Sex (male / female)	10 / 20	35 / 85
Side (right / left)	16 / 14	59 / 61
Mean age	70 (60–80)	69 (59–80)
Mean weight, kg	76 (53–110)	76 (46–106)
Charnley class (A / B / C)	17 / 11 / 2	64 / 52 / 4
Primary / secondary arthrosis	28 / 2	101 / 19
Mean HHS preoperatively	45 (14–83)	44 (19–82)

**Figure 1:**
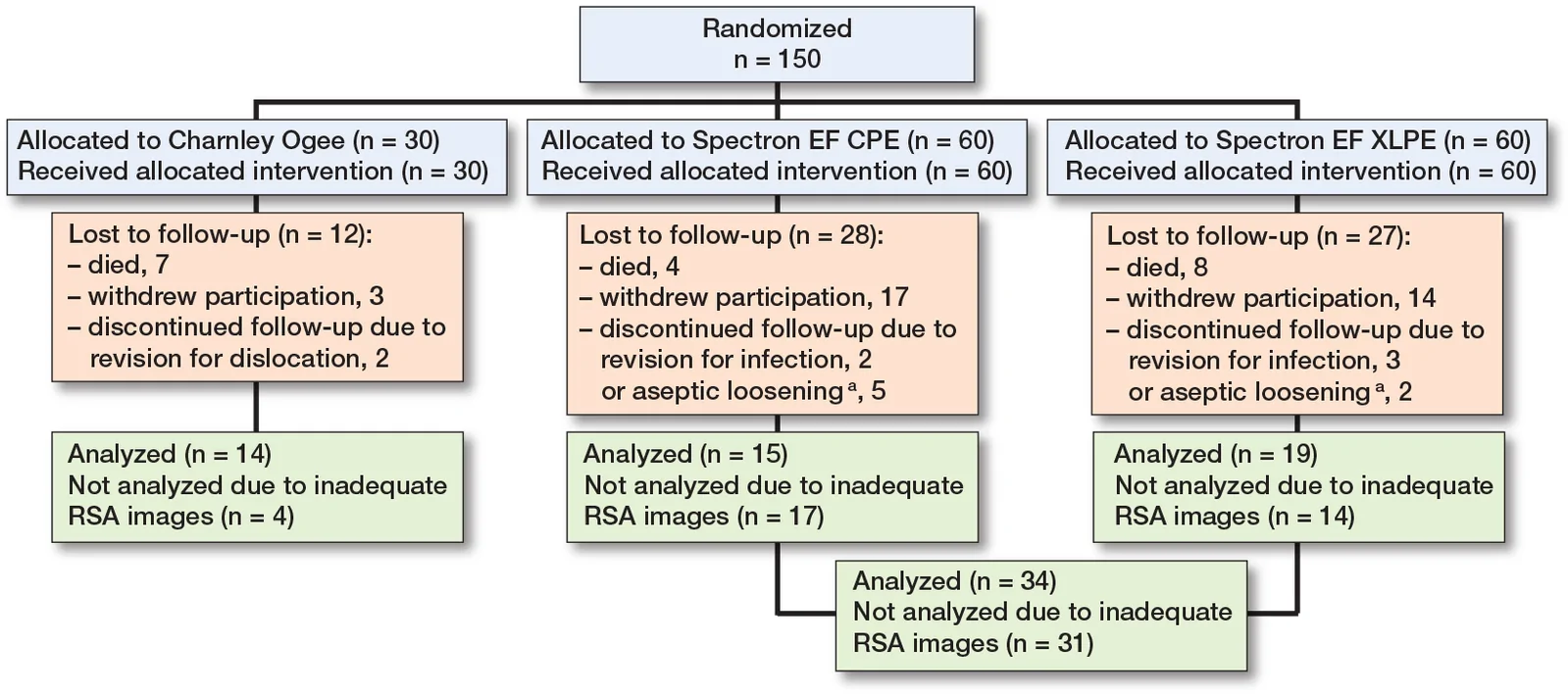
Cemented total hip arthroplasty with 2 different stems: flowchart of patients included and number of patients available for RSA analysis at 10 years, with reasons for exclusion at the 10-year follow-up.**^a^** Revision for aseptic loosening of the cup or cup and stem combined before the 10-year follow-up. No revisions were done due to aseptic loosening of the stem alone.

### RSA

The migration of the stems followed the same pattern shown at 2 years, and tended to migrate posteriorly, subside, and rotate into retroversion (Table 2). Movements in other directions were of lesser magnitude, with no significant differences between the groups, and the differences between the groups remained stable from 2 to 10 years.

**Table 2:** Migration (mm) and rotation (degrees) of the Charnley Ogee and the Spectron EF stems, estimates from linear mixed effects model, with 95% confidence intervals (CI). Number of individuals at each time point is provided (n), with the mean difference of the migration between the 2 stems at 10 years

Follow-up	Charnley Ogee		Spectron EF		Difference mean (CI)	P (interaction) ^a^
	n	mean (CI)	n	mean (CI)		
X-rotation: anterior tilt (+)/posterior tilt (–)						0.3
2 years	27	–0.07 (–0.29 to 0.15)	76	–0.10 (–0.22 to 0.03)	0.03 (–0.22 to 0.28)	
5 years	23	–0.11 (–0.34 to 0.12)	60	–0.16 (–0.29 to –0.02)	0.04 (–0.22 to 0.31)	
10 years	14	–0.08 (–0.35 to 0.19)	34	–0.25 (–0.42 to –0.08)	0.17 (–0.15 to 0.49)	
Y-rotation: retroversion (+)/anteversion (–)						0.2
2 years	27	2.3 (1.8 to 2.7)	76	0.67 (0.41 to 0.93)	1.6 (1.1 to 2.1)	
5 years	23	3.0 (2.5 to 3.4)	60	1.2 (0.91 to 1.5)	1.8 (1.2 to 2.3)	
10 years	14	3.2 (2.7 to 3.8)	34	1.5 (1.2 to 1.8)	1.7 (1.1 to 2.4)	
Z-rotation: adduction (+)/abduction (–)						0.3
2 years	27	–0.09 (–0.18 to 0.00)	76	–0.04 (–0.09 to 0.02)	–0.05 (–0.16 to 0.05)	
5 years	23	–0.16 (–0.26 to –0.06)	60	0.00 (–0.05 to 0.06)	–0.16 (–0.28 to –0.05)	
10 years	14	–0.12 (–0.23 to –0.00)	34	0.01 (–0.06 to 0.08)	0.04 (–0.06 to 0.15)	
X-translation: medial (+)/lateral (–)						0.08
2 years	27	0.05 (–0.02 to 0.11)	76	–0.03 (–0.07 to 0.00)	0.08 (0.00 to 0.15)	
5 years	23	0.01 (–0.06 to 0.08)	60	–0.01 (–0.05 to 0.03)	0.02 (–0.06 to 0.10)	
10 years	14	0.02 (–0.06 to 0.10)	34	0.08 (0.03 to 0.13)	–0.06 (–0.15 to 0.04)	
Y-translation: proximal (+)/distal (–)						0.1
2 years	27	–0.22 (–0.42 to –0.02)	76	–0.20 (–0.31 to –0.08)	–0.03 (–0.26 to 0.21)	
NN5 years	23	–0.33 (–0.54 to –0.11)	60	–0.36 (–0.49 to –0.23)	0.03 (–0.21 to 0.28)	
10 years	14	–0.32 (–0.57 to –0.06)	34	–0.72 (–0.88 to –0.56)	0.41 (0.11 to 0.71)	
Z-translation: anterior (+)/posterior (–)						0.9
2 years	27	–0.39 (–0.59 to –0.20)	76	–0.12 (–0.23 to –0.01)	–0.27 (–0.50 to –0.05)	
5 years	23	–0.53 (–0.71 to –0.30)	60	–0.34 (–0.47 to –0.22)	–0.16 (–0.40 to 0.08)	
10 years	14	–0.65 (–0.91 to –0.40)	34	–0.51 (–0.67 to –0.35)	–0.15 (–0.45 to 0.15)	

a P-value indicating whether the difference between stems varies over time. Null hypothesis is that the difference remains stable across the time period. Calculated by analyzing the interaction between articulation and time from the linear mixed effect model. Analysis including measures at 2, 5, and 10 years only.

The Charnley group had 3.2° (95% confidence interval [CI] 2.7–3.8) of retroversion at 10 years, compared with 1.5° (CI 1.2–1.8) for the Spectron EF group, with a mean difference of 1.7° (CI 1.1–2.4) (Figure 2).

**Figure 2:**
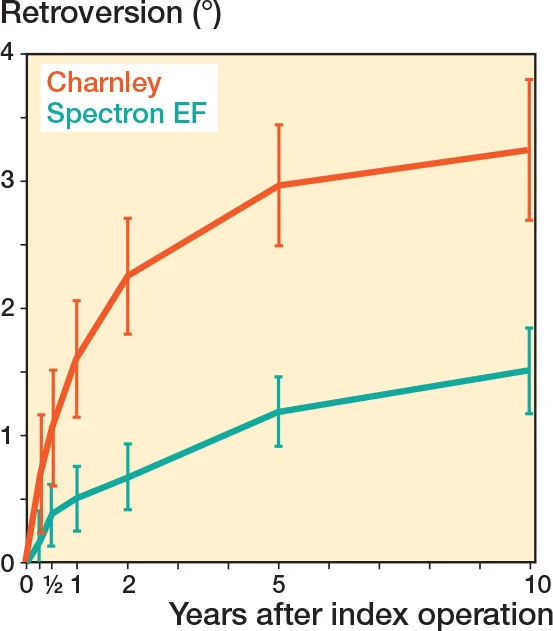
Mean retroversion (°) until the 10-year follow-up is shown for Charnley and Spectron EF cemented stems.

The mean posterior translation for the Charnley group was 0.65 mm (CI 0.40–0.91) and for the Spectron EF group 0.51 mm (CI 0.35–0.67), with a mean difference of 0.15 mm (CI –0.15 to 0.45) (Figure 3). Mean subsidence was 0.32 mm (CI 0.06–0.57) for the Charnley group and 0.72 mm (CI 0.56–0.88) for the Spectron EF group, with a difference of 0.41 mm (CI 0.11–0.71) (Figure 4).

**Figure 3:**
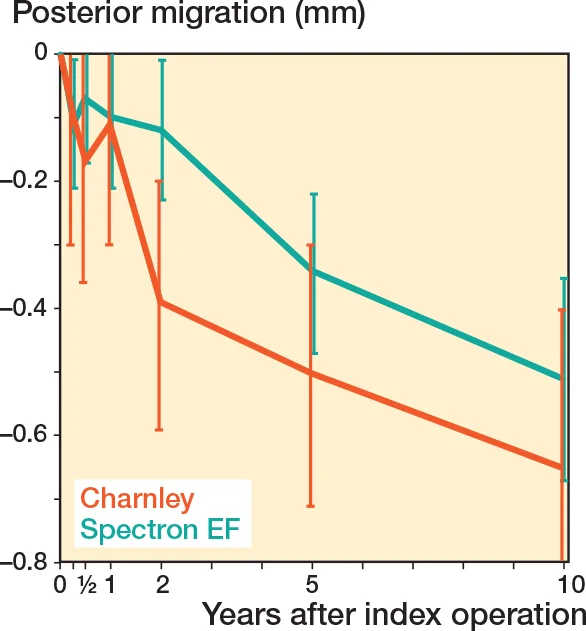
Mean posterior migration (mm) (along the z-axis) until the 10-year follow-up is shown for Charnley and Spectron EF cemented stems.

**Figure 4:**
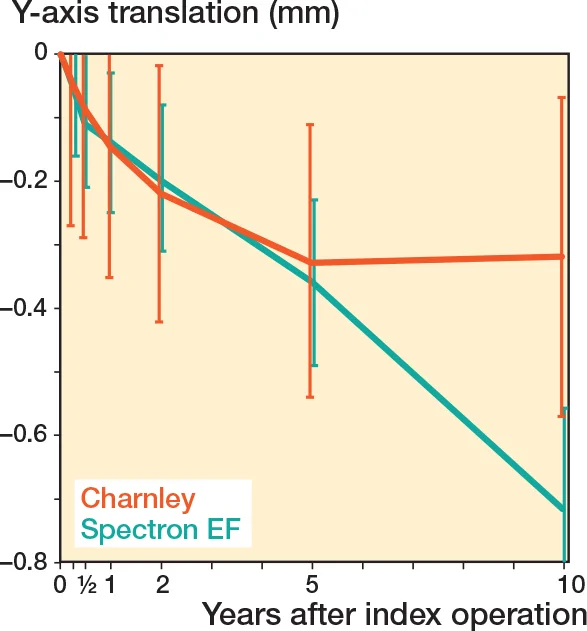
Mean translation along the y-axis (mm) until the 10-year follow-up is shown for Charnley and Spectron EF cemented stems. Distal translation/subsidence has values < 0.

Within the Spectron EF group the stems articulating with XLPE had greater subsidence at 0.92 mm (CI 0.71–1.14) compared with stems articulating with CPE with 0.48 mm (CI 0.25–0.72), with a mean difference of 0.44 mm (CI 0.12–0.76). There were no differences in migration in any other direction (Supplementary Table S1, Supplementary Figures S3–S5).

### Revisions

7 THAs were revised due to aseptic loosening, and all were within the Spectron EF group, before the 10-year RSA follow-up. Of these, 5 were due to loosening of the cup alone, while 2 cases had loosening of both the cup and stem, with 1 combined with a CPE cup, and 1 with an XLPE cup. Neither of these 2 stems had available RSA migration data at 2 years. However, the loose stem with CPE cup had retroversion of 2.4°, and subsidence of 0.01 mm at 6 months.

### Clinical outcomes

Both groups had a similar increase in HHS from preoperatively until the 10-year follow-up. In the Charnley group the HHS increased from a preoperative 41 (SD 15) to 86 (SD 19) at 10 years, while the Spectron EF stems had a preoperative HHS of 45 (SD 13), and increased to 86 (SD 15). There was no statistically significant difference between the groups.

## Discussion

In the present study we aimed to compare the migration of 2 different stems in cemented THA over 10 years using RSA. For our primary outcome we found similar migration patterns for the 2 stems. Both migrated posteriorly, subsided, and rotated into retroversion, following the pattern found at 2 years. Migration in other directions was minor. They differed in outcome by the Charnley stems having higher retroversion and less subsidence compared with the Spectron EF stems. For our secondary aims, we found that the use of XLPE or CPE in the articulations did not influence the migration for the Spectron EF stem, other than XLPE articulating stems having greater subsidence The clinical outcomes (HHS) were similar between the 2 stems.

At 2 years, the pattern of migration was consistent with what we found after 10 years [11]. Still, there was a significant difference in retroversion for the Charnley stems. However, the significant difference in posterior migration found at 2 years’ follow-up was not evident now. This could partly be explained by the decrease in available subjects, and loss of statistical power. Conversely, the data indicates that most of the rotational change, particularly retroversion, but also migration in other directions, occurs within the first 2 years, after which the remaining stems appear relatively stable between 2 and 10 years. However, our data suggests that the Spectron EF stem continues to subside at a relatively even rate. All 7 revised THAs were within the Spectron EF group.

A previous RSA study of the Spectron EF stems found that the degree of retroversion and subsidence at 2 years could predict later loosening [17]. This could perhaps be because it fails to completely bond to the cement mantle with subsequent continuous subsidence. It is important to note that this increased subsidence could be attributed to a few Spectron EF stems with higher levels of migration, as visualized in Supplementary Figure 7. The 4 Spectron EF stems with greatest subsidence at 10 years all had HHS between 82 and 100 at 10-year follow-up, increasing from between 33 and 53 preoperatively. None of these stems was considered as loose when evaluating radiographs at 10 years, but 1 was later revised for aseptic loosening after the end of RSA follow-up. In addition, 3 of the 4 stems with greatest subsidence articulated with XLPE, which could explain the difference in subsidence within the Spectron EF group as well.

Still, with this increased subsidence for the Spectron EF stem, a previous long-term RSA study of the well-performing composite beam Lubinus SPII stem showed similar levels of subsidence [18]. Furthermore, both stems had less subsidence than the taper-slip stem Exeter in comparable RSA-studies [19,20].

Due to poor long-term results for the Spectron EF stem in registry and clinical studies we anticipated that perhaps the Spectron EF stem would reach levels of retroversion similar to the Charnley stem from 2 years [10,21]. This was not the case. A possible explanation for this could be differences in the surface and shape of the stems. The Charnley stem has a smoother surface and oval shape, while the Spectron EF stem has a proximal roughened surface and a rectangular shape. Thus, the Charnley stem could be more susceptible to rotation. When considering the difference in surface, the smoother Charnley stem would release fewer cement particles, and have less subsequent cellular response, compared with the rough Spectron EF stem, which may produce more cement particles under movement. The Norwegian Arthroplasty Register has reported higher revision rates for the Spectron EF stem compared with the Charnley stem (RR = 6.1) [10]. The rough proximal surface intended to improve fixation may instead lead to debonding or particle generation, supporting earlier findings, and explain the increased subsidence of the Spectron EF stems from 2 to 10 years [21,22].

The Reflection All-Poly CPE cup had significant levels of PE wear in our previous study, with acetabular osteolysis and cup loosening, over the 10-year period [7]. We theorized that this perhaps also could lead to femoral osteolysis and affect the stem loosening. However, the migration of the stems with high-wear CPE cups did not differ from the migration of stems coupled with low-wear XLPE cups, with even XLPE articulating stems having more subsidence. This finding suggests that PE wear was not the cause of migration and subsequent loosening. From a retrieval analysis of the Spectron EF stem it was hypothesized that micromotion of the rough stem may lead to the release of cement particles, eventually leading to osteolysis and loosening [22]. These micromovements may be of smaller magnitude than the ones we measured with RSA in the present study. Another study argued, in similar fashion for the Charnley stems, that the release of PE particles could not be the reason for aseptic stem loosening [23]. It could also be that the loose stems that were revised together with the cup had high levels of migration which remain undetected because these stems were excluded from further analysis after revision. Furthermore, when cup revisions were performed, surgeons may tend to also revise the paired Spectron EF stem, after testing fixation of the stem during surgery. In all cases of stem revision, the cup was also revised.

### Limitations

At 10 years a substantial number of stems were excluded from RSA analysis. This point was discussed in the reporting of the wear and migration at 10 years for the acetabular cups from the same material [7]. The same number of patients were lost to follow-up due to withdrawal of participation or death, as well as revisions for other reasons. The additional losses were most likely attributable to the cone containing a tantalum marker positioned in the neck of the Spectron EF stem. These tended to loosen, making the CN and ME numbers too high and RSA examinations were excluded. In addition, the relatively low number of stems followed fully until 10 years gives the possibility of results being influenced by individual stems with high migration, such as could be the case of the increased subsidence for the XLPE articulating Spectron EF stems. Furthermore, only intact stems were investigated, meaning additional differences between the groups could be hidden in the revised hips. Some of this potential attrition bias can be compensated for by using the linear-mixed model; all the available data was included, even from cases that did not have measurements at all data points. Detection bias may have occurred, as surgeons performing follow-up assessments might have become aware of the articulation used.

### Conclusion

We found similar patterns of migration for the Charnley and Spectron EF stems at 10 years. The discernible differences were in increased retroversion for the Charnley stems and increased subsidence for the Spectron EF stems, with most of the increased subsidence being within the XLPE articulating stems. Otherwise, the choice of polyethylene cup material did not affect Spectron EF stem migration. Clinical results were similar for both stems.

### Supplementary data

Table S1 and Figures S1–S7 are available as supplementary data on the article home page, doi: 10.2340/17453674.2026.46836

## Supplementary Material



## Notes

HGJ: data collection, analysis, and writing. GH, TK, OF: planning, data collection, and revision of the manuscript. AMF, AA, and KH: data collection, analysis, and revision of the manuscript. MB, and KI: data collection and revision of the manuscript. SHLL: data analysis and revision of the manuscript. PJH: writing and revision of the manuscript, figures.

The authors would like to thank the surgeons at Bergen Hospital Trust (Helse Bergen HF) and the subject participants for their support in conducting this research. Special thanks to Terje Stokke (Radiology Department, Haukeland University Hospital) for producing all the RSA radiographs.

Handling co-editors: Bart Pijls and Robin Christensen

*Acta* thanks Marc J Nieuwenhuijse and Christiaan Righolt for help with peer review of this manuscript.
