# Supplementary material for: A randomized study on migration of the Spectron EF and the Charnley flanged 40 cemented femoral stems: secondary analysis of a randomized controlled trial using radiostereometric analysis (RSA) with 10 years’ follow-up
Source: Acta Orthop. 2026 Nov 12;97:814–20. doi: 10.2340/17453674.2026.46836 (PMC13757272; doi:10.2340/17453674.2026.46836)
Supplement: Supplementary file 1 [file ActaO-97-46836-s1.pdf]

Supplementary data

Supplementary Table S1. Cemented total hip arthroplasty with 2 different stems: Migration and rotation between the stems and bone, estimates from linear mixed effects model with the mean difference of the migration between the 2 PE types within the Spectron EF group at 10-years

Follow-up	Charnley Ogee	Spectron EF CPE	Spectron EF XLPE	Difference within Spectron EF
X-rotation: Anterior tilt (+) / posterior tilt (–)				
2 years	–0.07 (–0.29 to 0.15)	0.01 (–0.16 to 0.19)	–0.22 (–0.40 to –0.04)	–0.23 (–0.48 to 0.02)
5 years	–0.11 (–0.34 to 0.12)	–0.11 (–0.29 to 0.08)	–0.22 (–0.42 to –0.02)	–0.11 (–0.30 to 0.29)
10 years	–0.08 (–0.35 to 0.19)	–0.16 (–0.41 to 0.09)	–0.35 (–0.58 to –0.12)	–0.19 (–0.53 to 0.15)
Y– rotation: Retroversion (+) / anteversion (–)				
2 years	2.3 (1.8 to 2.7)	0.66 (0.21 to 1.1)	0.68 (0.22 to 1.1)	0.02 (–0.63 to 0.66)
5 years	3.0 (2.5 to 3.4)	1.2 (0.70 to 1.7)	1.2 (0.63 to 1.1)	–0.03 (–0.74 to 0.68)
10 years	3.2 (2.7 to 3.8)	1.4 (0.71 to 2.1)	1.6 (0.95 to 2.2)	0.17 (–0.74 to 1.1)
Z–rotation: Adduction (+) / abduction (–)				
2 years	–0.09 (–0.18 to 0.00)	–0.09 (–0.16 to –0.01)	0.02 (–0.06 to 0.09)	0.10 (–0.00 to 0.21)
5 years	–0.16 (–0.26 to –0.06)	–0.05 (–0.13 to 0.03)	0.06 (–0.02 to 0.14)	0.11 (–0.00 to 0.22)
10 years	–0.12 (–0.23 to –0.00)	–0.08 (–0.18 to 0.03)	0.09 (–0.00 to 0.19)	0.17 (0.03 to 0.31)
X–translation: Medial (+) / lateral (–)				
2 years	0.05 (–0.02 to 0.11)	–0.04 (–0.09 to 0.01)	–0.02 (–0.07 to 0.03)	0.03 (–0.05 to 0.10)
5 years	0.01 (–0.06 to 0.08)	–0.03 (–0.08 to 0.03)	0.01 (–0.05 to 0.06)	0.03 (–0.05 to 0.12)
10 years	0.02 (–0.06 to 0.10)	0.03 (–0.04 to 0.10)	0.12 (0.05 to 0.19)	0.09 (–0.01 to 0.19)
Y–translation: Proximal (+) / distal (–)				
2 years	–0.22 (–0.42 to –0.02)	–0.15 (–0.31 to 0.01)	–0.24 (–0.41 to –0.08)	–0.10 (–0.33 to 0.14)
5 years	–0.33 (–0.54 to –0.11)	–0.27 (–0.45 to –0.10)	–0.46 (–0.65 to –0.27)	–0.19 (–0.44 to 0.07)
10 years	–0.32 (–0.57 to –0.06)	–0.48 (–0.72 to –0.25)	–0.92 (–1.14 to –0.71)	–0.44 (–0.76 to –0.12)
Z–translation: Anterior (+) / posterior (–)				
2 years	–0.39 (–0.59 to –0.20)	–0.13 (–0.29 to 0.03)	–0.11 (–0.28 to 0.05)	0.01 (–0.51 to –0.01)
5 years	–0.53 (–0.71 to –0.30)	–0.31 (–0.48 to –0.14)	–0.38 (–0.56 to –0.20)	–0.07 (–0.32 to 0.07)
10 years	–0.65 (–91 to –0.40)	–0.47 (–0.71 to –0.23)	–0.54 (–0.76 to –0.32)	–0.07 (–0.39 to 0.25)

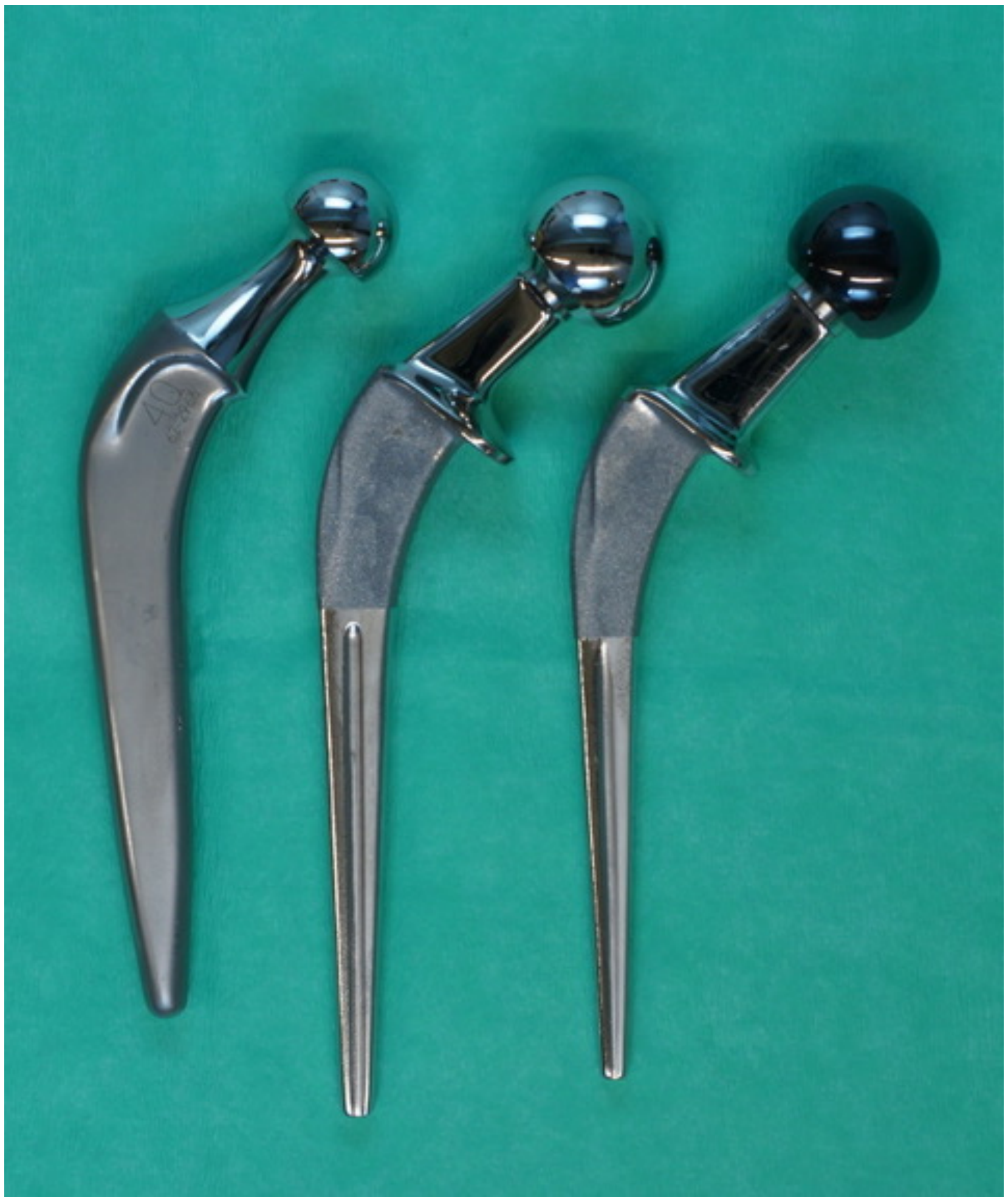

Supplementary Figure S1. Cemented total hip arthroplasty with 2 different stems**:** Picture of the 2 stems used, with both head materials for the Spectron EF. Charnley (left), Spectron EF with CoCr head (middle) and Spectron EF with Oxinium head (right). Image credit to PJH.

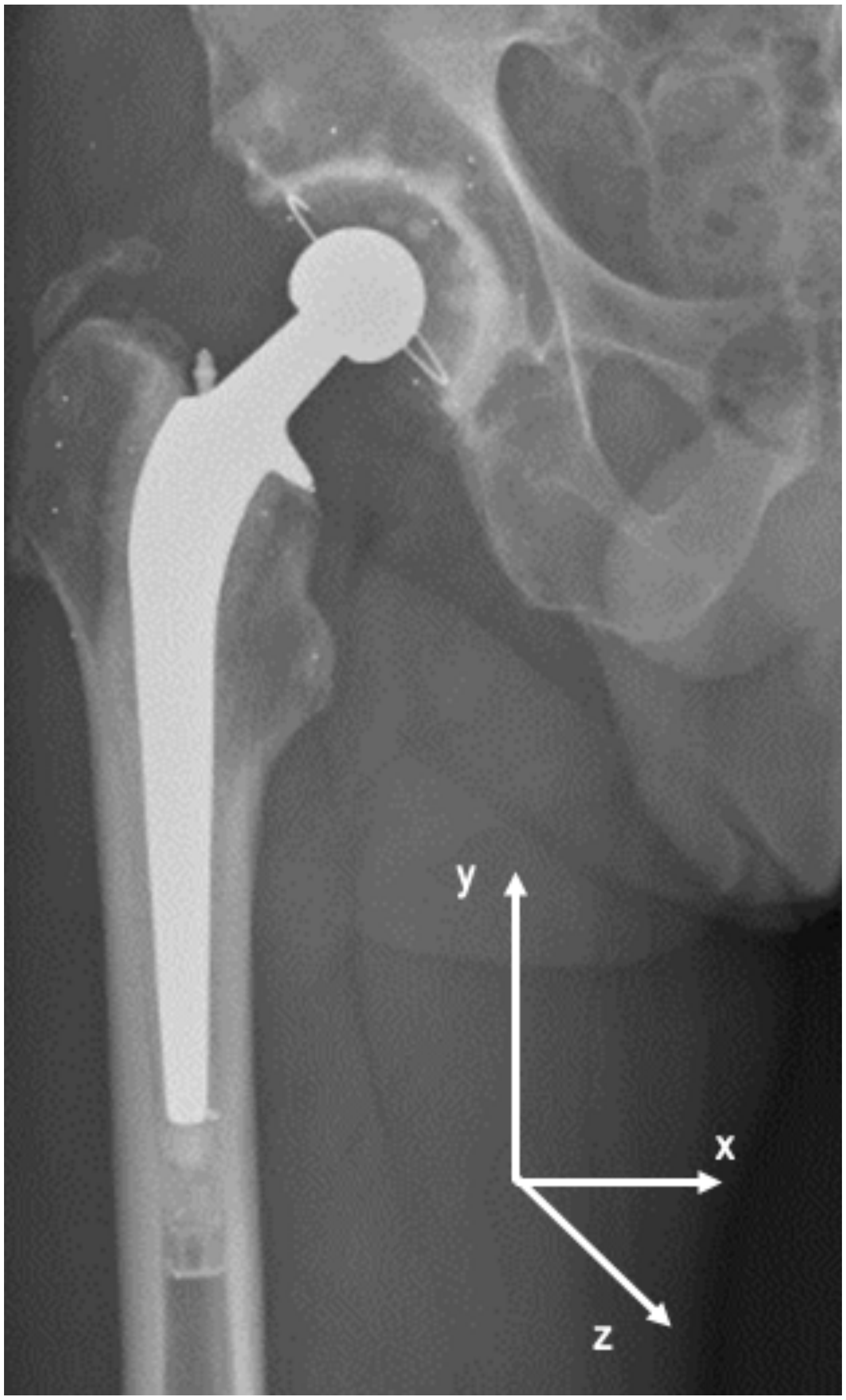

Supplementary Figure S2. Radiograph showing the tantalum markers for the Spectron EF stem, including the impacted cone. Also demonstrating the directions for the x, y and z axes.

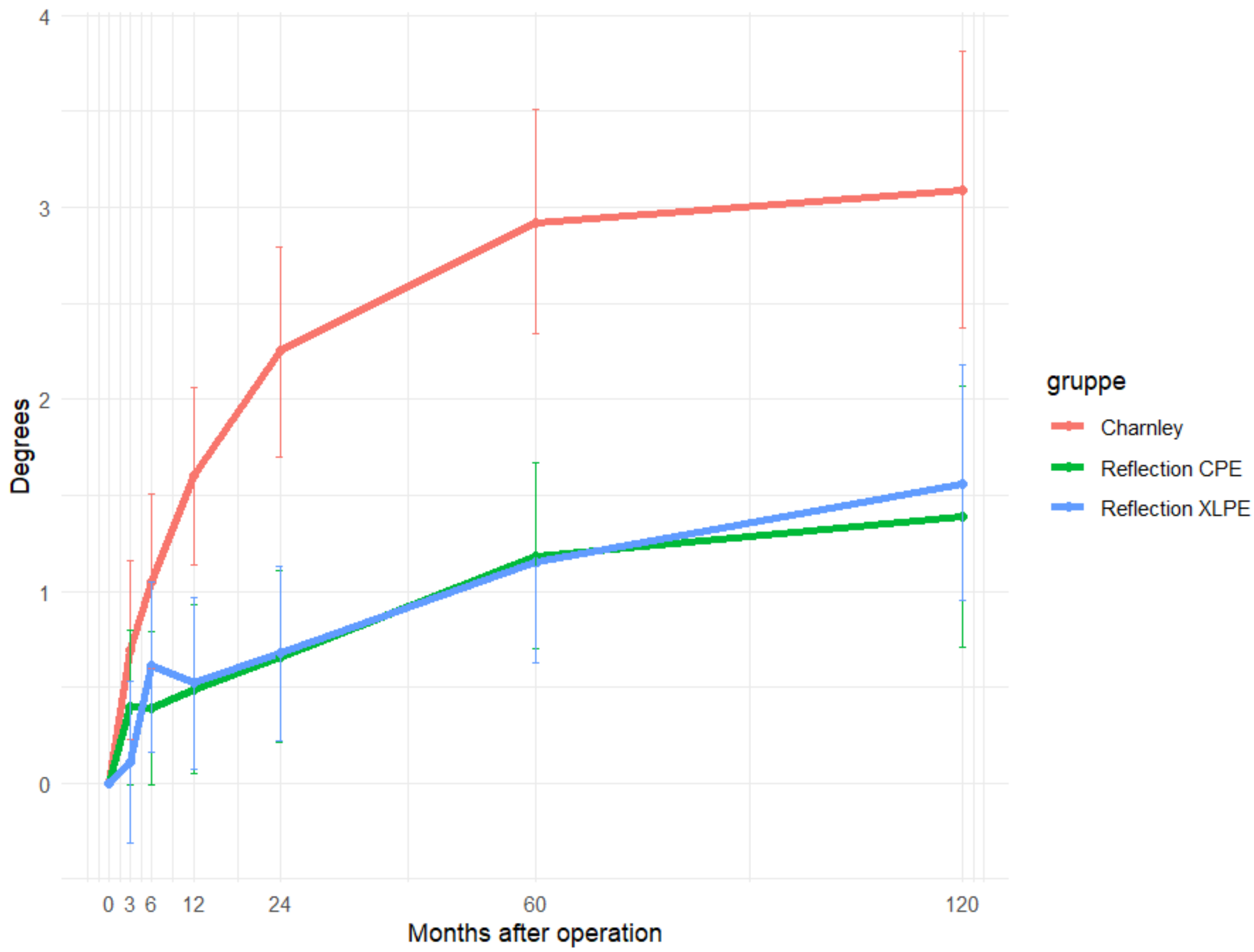

Supplementary Figure S3. Cemented total hip arthroplasty with 2 different stems: Graph showing mean retroversion (°) until the 10-year follow-up for the 2 stems, with the Spectron EF divided into CPE and XLPE.

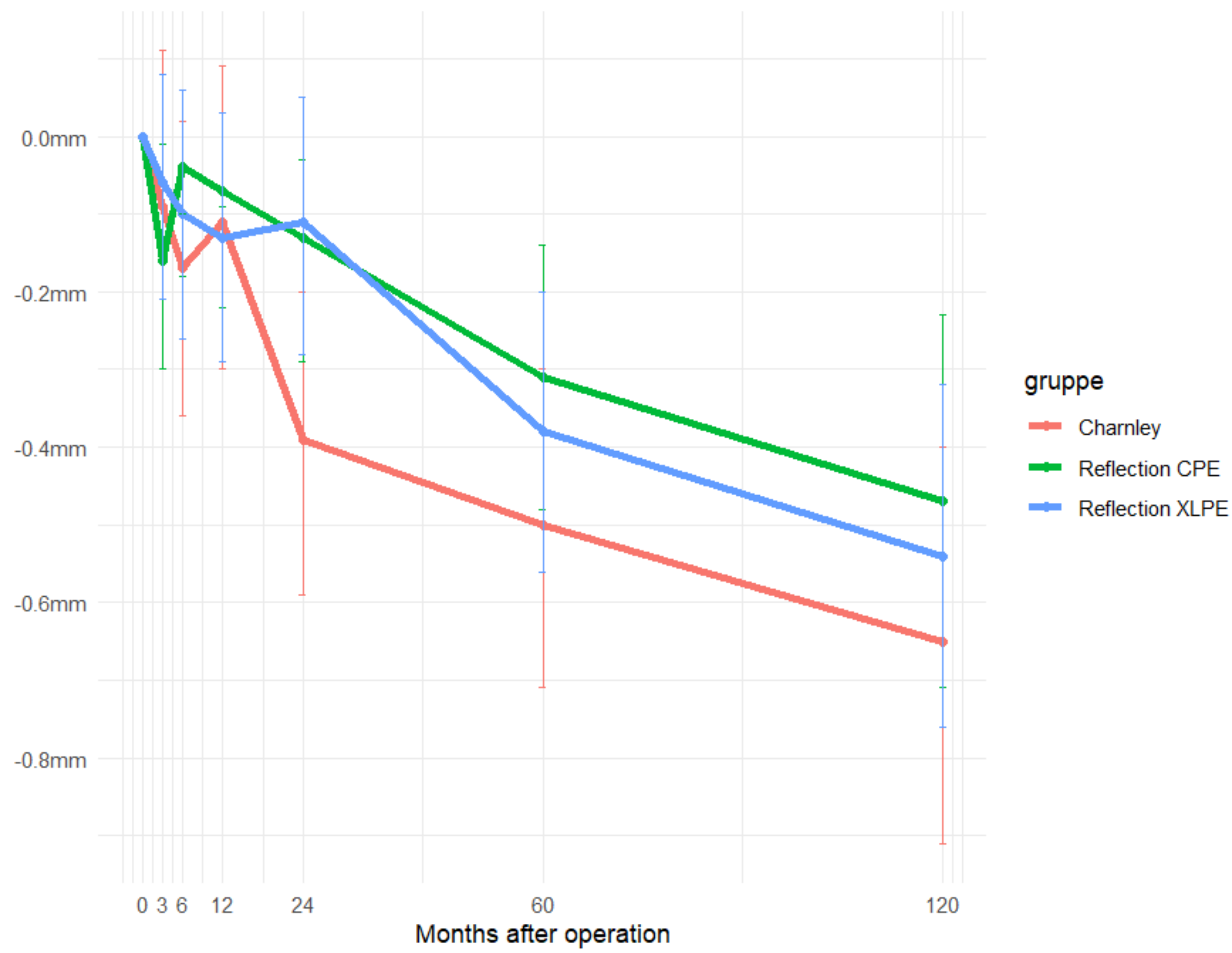

Supplementary Figure S4. Cemented total hip arthroplasty with 2 different stems: Graph showing mean posterior migration in mm (along the z-axis) until the 10-year follow-up for the 2 stems, with the Spectron EF divided to CPE and XLPE

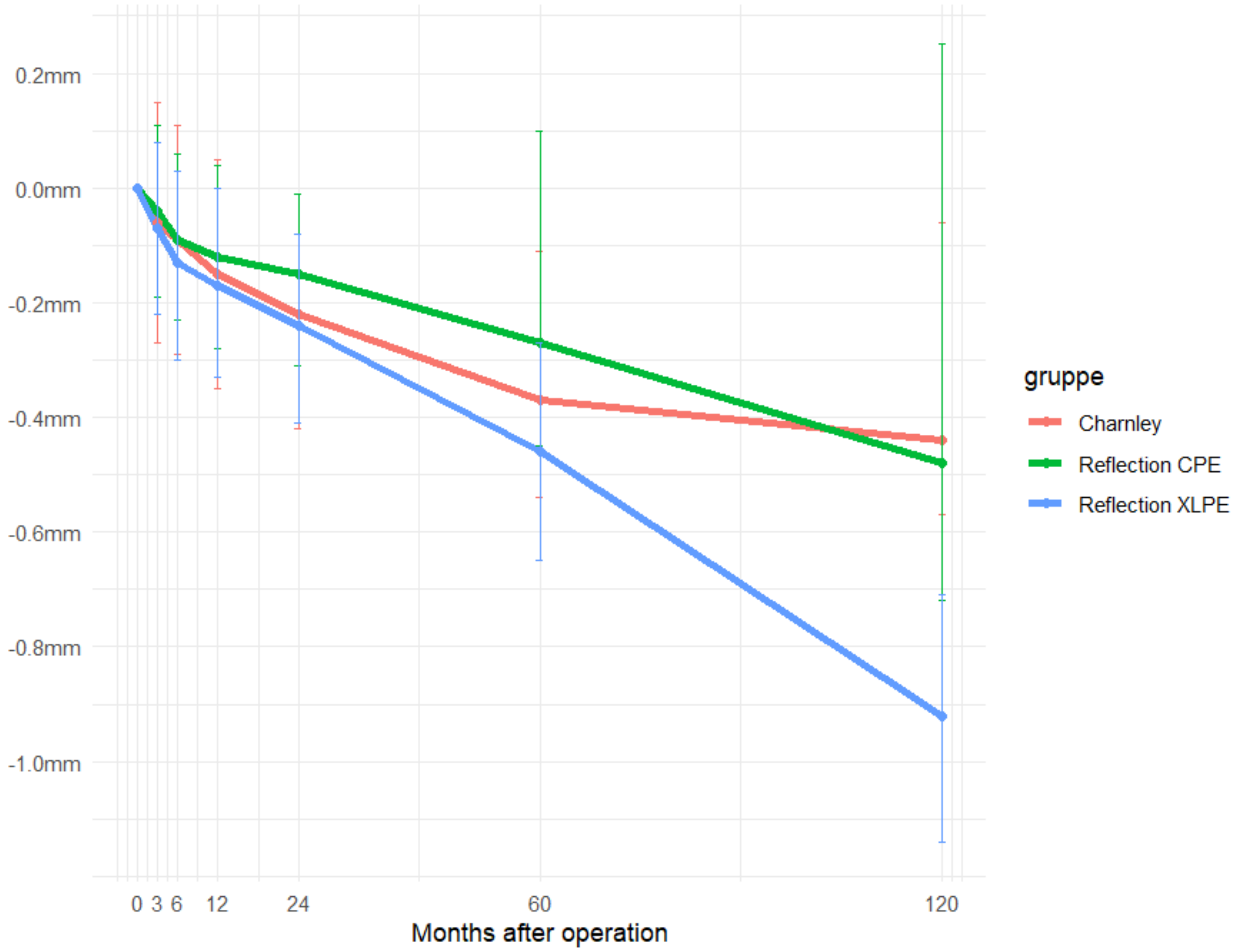

Supplementary Figure S5. Cemented total hip arthroplasty with 2 different stems**:** Graph showing mean translation along the y-axis in mm (distal translation, or subsidence, has values <0), until the 10-year follow-up for the 2 stems with the Spectron EF divided in CPE and XLPE.

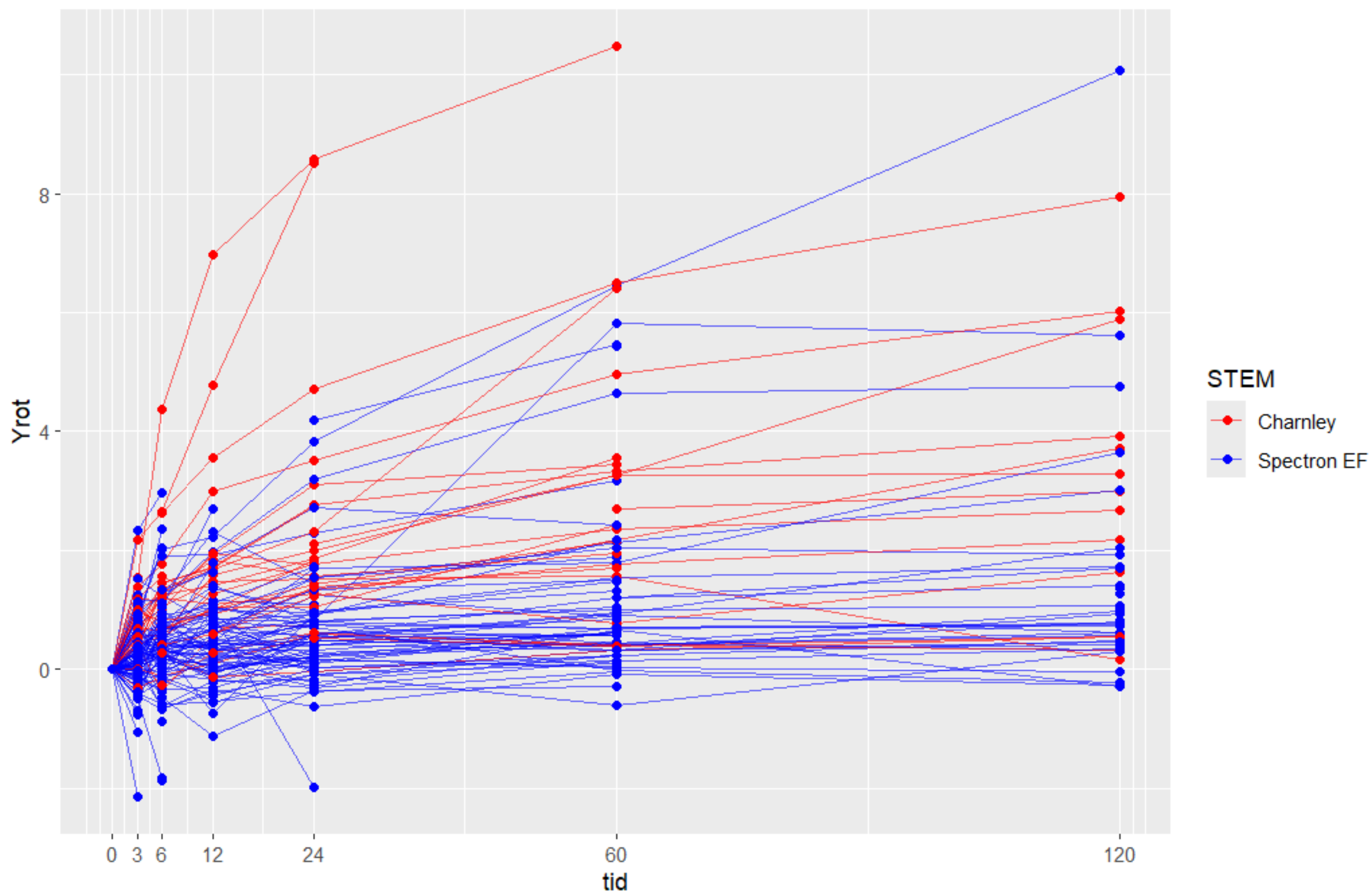

Supplementary Figure S6. Single-line plot showing retroversion for patients with available RSA measurements at each time point.

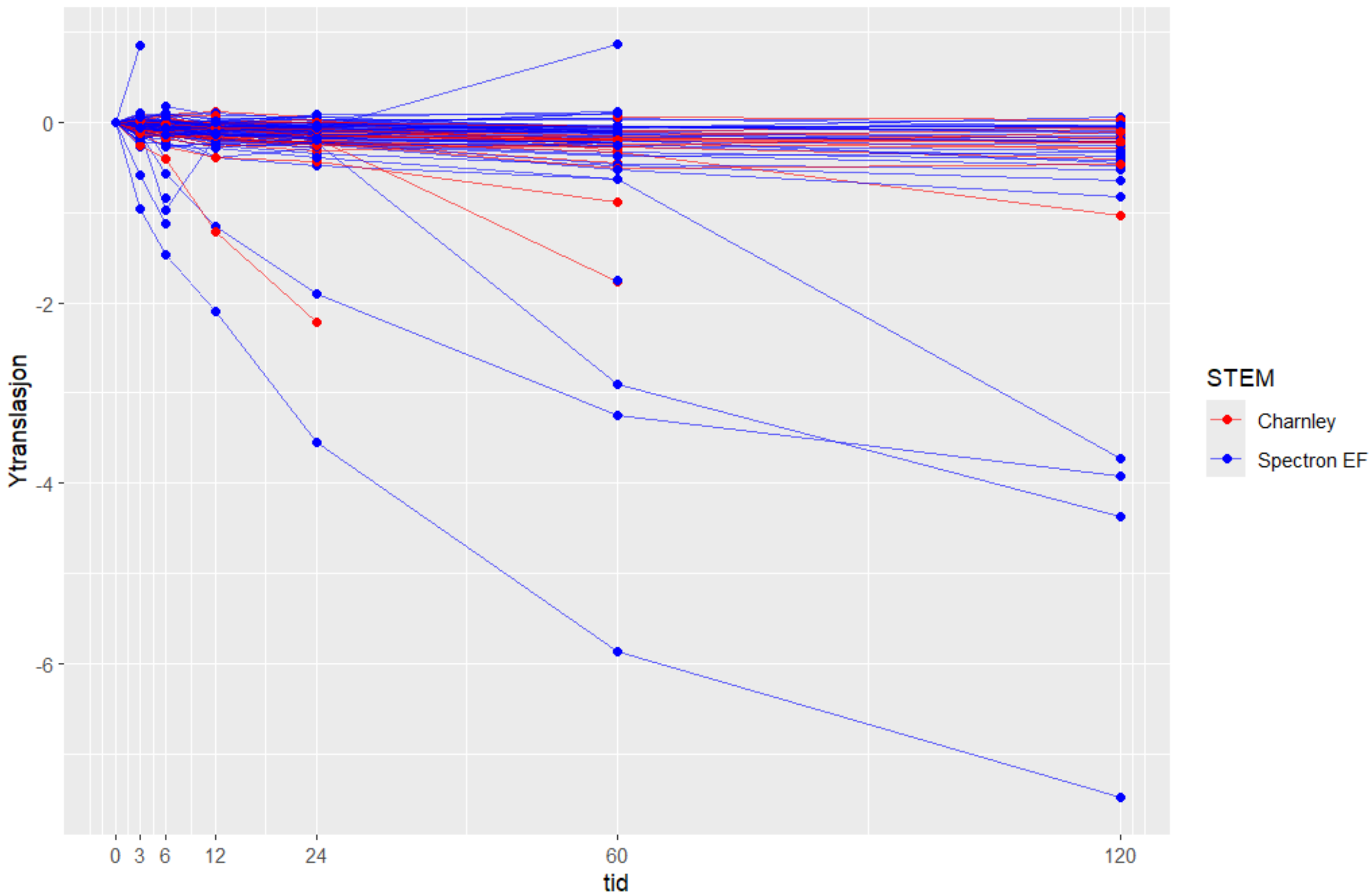

Supplementary Figure S7. Single-line plot showing subsidence for patients with availiable RSA-measurments at each time point.
